# Effect of wooden floorboards on the vibration of timber floor

**DOI:** 10.1038/s41598-023-50015-5

**Published:** 2024-01-08

**Authors:** Osama A. B. Hassan

**Affiliations:** Department of Science and Technology, Linköping University, 601 74 Norrköping, Sweden

**Keywords:** Environmental sciences, Engineering

## Abstract

This study is aimed at investigating those parameters related to timber flooring that can affect the acceptability of vibration behaviour of a timber floor in a residential building in view of the criteria stated in Eurocode EC5. The timber floor investigated is made of OSB/3 floorboards and timber joists. The parameters that are investigated in this study are thickness of flooring, floor joist span, joist spacing and connection of floorboards to the joists. In this context, two cases are considered. First: the flooring is nailed or screwed to the joists and no composite action or interaction is obtained between joists and floorboards. Second: the flooring is glued sufficiently to the joists and full interaction is obtained. The result suggests that glued floorboards perform much better with respect to natural frequency, static deflection and peak floor velocity than nailed or screwed floorboards. In almost all cases of glued floorboards, the result complies fully with the Eurocode 5 design vibration requirements. However, as floor lengths increase, the static deflection will increase beyond the allowable limit, especially for relatively thin floor panels and relatively widely spread joists. For both cases, increasing floorboards thickness and decreasing the joist span by adding more beams can yield even better results to satisfy the requirement of vibration comfort.

## Introduction

Floor vibration is one of the requirements for design check to be considered for structural design. Human-induced vibrations in timber structures can cause unacceptable discomfort to users, especially due to low frequency walking-induced vibrations. Floor vibrations can even, in worst cases, impair the functioning or serviceability of the structure. The footfall- induced vibration in timber floors can have two types of components, low-frequency components due to step frequency and its harmonics and higher-frequency components due to heel excitation of the floor. The properties that influence the adequacy of a timber floor with respect to vibrations are mainly the bending stiffness of the structure, area of the floor and material damping. Especially for longer spans and lighter structure, timber floors possess relatively low natural frequencies and high resonant behaviour^1^.

To evaluate the acceptability of floor vibrations in timber floors, different requirements and recommendations are presented in design standards, depending on the adapted building code. The common denominator between these codes is the criteria of natural frequency, deflection, and peak velocity of the floor due to human footfalls compared to recommended limiting values. This paper investigates the vibration in pure timber floors in accordance with the design method for vibrational serviceability of timber floors proposed in Eurocode 5^2^.

Several studies to investigate the vibration of timber floors have been published. Bernard investigated the vibration in timber floors built with I-timber beams^3^. Hassan and Girhammar^1^ assessed the floor acceptability of two composite structures with respect to composite action. Zhang, et al.^4^ compared vibrational comfort criteria for different European countries in which the timber floors are constructed with metal web joists using plywood flooring. Alexander et al.^5^ investigated the effect of damping in lightweight timber floors. Huang et al.^6^ studied the effect of boundary conditions of Cross-Laminated Timber (CLT) floor on the vibration response. Abd Ghafar et al.^7^ assessed the vibration behaviour due to walking loads in floors constructed using glued laminate (glulam) timber. Weckendorf et al.^8^ analysed the vibration serviceability performance of timber floors as determined by Eurocode 5 and found that reasonable results can be obtained for residential floors with spans of up to 6 m. Zhoua et al.^9^ performed a vibration test of a wooden floor structure for a gymnasium with respect to different types of floor structures. Hamm et al.^10^ investigated floor vibration and concluded that non-bearing partition walls have influence on the vibration behaviour. Hu^11^ presented some practical measures to control vibrations in timber floors. A state-of-the-art review of the recent vibration issues in timber structures can be found in Aloisio et al.^12^.

Often in the evaluation of floor performance against vibration, the effect of flooring is neglected, particularly in the calculation of natural frequencies and deflection, since the joists have normally more influence on the results than the flooring properties. However, the floorboards’ effect can be evident, especially for higher floor dimensions. The article will examine this case using the OSB as flooring material. OSB (Oriented Strand Board) is increasingly gaining attention in modern timber houses for its suitability as floorboards. Compared to plywood, it is cheaper and offers comparable flexural strength. Moreover, OSB is stronger than plywood in shear^14^, which makes it suitable in wooden I-beams that are affected by shear concentrations. With respect to sustainability in terms of durability and environmentally friendly material, it is claimed that OSB is be more eco-friendly than most plywood materials^13^. Moreover, in the manufacturing of OSB, there is no need to harvest large old-growth trees, since it can be made from smaller trees. Technically, OSB is synthetic wooden panels made of layers of wood strands or chips that are compressed together with construction adhesives (e.g. wax, phenol and resin) under high pressure and heat to form flat panels ready to install, ‘in an identical way to that used for plywood at the building site'. As with plywood, the OSB board has a clear main direction. The chips near the panel surface lie in the longitudinal direction (long axis) of the panel, which have greater strength, while the chips in the core layer (middle part) lie perpendicular to the longitudinal direction. In practice, OSB boards are available in types 1, 2, 3 and 4. Type OSB/1 is for general use and for interior decoration in dry premises. Type 4 is the heaviest and most moisture resistant. Type OSB/3 can be used in various situations in load-bearing constructions.

This study is aimed at investigating those parameters related to floorboards that can affect the acceptability of vibration behaviour in view of the criteria stated in Eurocode EC5^2^. A parametric study is carried out to investigate how the thickness of floorboards, floor joist span, spacing between joists, and connection of floorboard to the joists is affecting the adequacy of wood-framed floors for vibration comfort.

## Methodology

**Figure 1:**
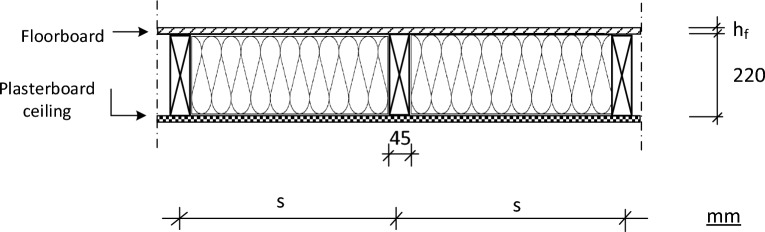
Timber floor construction.

The floor construction investigated is shown in Fig. 1. It is composed of timber beams, oriented strand board (OSB/3) nailed, screwed or glued on its upper edge, plasterboard ceiling at its bottom edge for decoration and to increase fire resistance, and stone wool, used for soundproofing and heat insulation. This floor system is typical for a single-family house. The structural design requirements of the floor system are based on the European standard Eurocode 5^2^. Timber joists are made of solid structural timber with a strength class C30, dimensions 45 × 220 (standard section size), and are spaced at a centre distance of 600 mm or 400 mm. The floor system is considered as simply supported on all four sides.

Different thicknesses of floorboards (*h*_f_) are chosen for the study, as follows: 18 mm, 20 mm, 22 mm, 25 mm.

To investigate the effect of a floor joist span on the adequacy of floor to vibration, the dimensions of the floor structure (*B* x *L*), where *L* is the floor span between supports (joist span) and *B* is the floor width, are chosen as follows: 5 m × 3.7 m; 5 m × 4 m; 5 m × 4.5 m; 5 m × 5 m. These dimensions represent typical room areas in timber houses. The spacing between joists is further investigated with *s* = 600 mm and *s* = 400 mm.

To investigate the effect of connection of floorboard to the joists, two cases are considered. First: the floorboards are nailed or screwed to the joists and no composite action or interaction is obtained between joists and floorboards. Second: the floorboards are glued sufficiently to the joists and full interaction is obtained.

The material and geometrical properties of the joists and floorboards are chosen so that the structural requirements (moment, shear, bearing pressure, deformation) are first satisfied for all the investigated cases, according to the EC5^2^ for a typical residential building in which the imposed load (characteristic variable action) belong to category A (2 kN/m^2^) and the timber structure is in service class 2. Table 1 shows material properties of the floor construction.

**Table 1:** Material properties of the floor structure.

Floorboard	Property
Oriented strand board (OSB/3) (Main chip direction is parallel to the joist)	${E}_{0,mean}=4930 MPa ( {h}_{f}>6-25 mm)$ ${\rho }_{mean}=550 {\text{kg}}/{{\text{m}}}^{3}$
Joist C30	${E}_{0,mean}=12000 {\text{MPa}}$ ${\rho }_{mean}=460 {\text{kg}}/{{\text{m}}}^{3}$
Plasterboard ceiling Mineral wool	Self-weight *g*_*k*_ = 0.2 kN/m^2^ Self-weight *g*_*k*_ = 18 kg/m^3^

${E}_{0,mean}$= Mean modulus of elasticity parallel to the grain. ${\rho }_{mean}$= Mean density.

The floor mass, *m*, is based on how the characteristic permanent action on the floor varies with respect to floor thickness and joist spacing and is estimated according to Table 2. Technically, the stone wool (height 220 mm) accounts for an extra mass, approx. 3.7 kg/m^2^ for *s* = 0.6 m and 3.5 kg/m^2^ for *s* = 0.4 m. The mass values shown in Table 2 represent typical design masses for most floor constructions in single-family houses, although construction details can vary.

**Table 2:** Calculated masses of floor constructions.

Thickness, *h*_f_ (mm)	Mass,* m* (kg/m^2^)	Mass, *m* (kg/m^2^)
	Centres of timber joists, *s* = 600 mm	Centres of timber joists, *s* = 400 mm
18	42	46
20	43	47
22	44	48
25	46	50

For each case, a design check will be carried out to assess if the vibration behaviour of the floor will be acceptable. Consequently, conclusions will be drawn based on the results of parametric study to guide the designers to different practical solutions that can be considered to make the timber floor construction adequate for vibration as a part of fulfilling serviceability requirements.

## Vibration criteria

Eurocode 5^2^ describes a procedure for evaluating the vibration characteristics of residential floors. Certain requirements must be satisfied in order not to cause unacceptable discomfort to users, as follows.

- The fundamental (natural) frequency of vibration of a rectangular residential floor *f*_1_ should be normally greater than 8 Hz, since humans are most sensitive to vibrations in the frequency range up to 8 Hz^14^. For *f*_1_ ≤ 8 Hz, special investigation should be performed. For a simply supported plate along its four edges with timber beams having a span* L*, ${f}_{1} ({\text{Hz}})$ can be obtained as:
  1 $${f}_{1}=\frac{\pi }{2{L}^{2}}\sqrt{\frac{{\left(EI\right)}_{L}}{m}} ({\text{Hz}})$$

where *L* is the span of the floor in the direction of the load-bearing beams (joist span) (m), (*EI*)_*L*_ is the equivalent plate bending stiffness of the floor about an axis perpendicular to the beam direction (the stiffer direction) (Nm^2^/m), and *m* is the mass per unit area (kg/m^2^).

- The static deflection of the floor can be expressed as:
  2 $$\frac{w}{ F}\le a ({\text{mm}}/{\text{kN}}),$$

where *w* is the instantaneous maximum vertical deflection cased by a concentrated static force *F* = 1 kN, applied normally at floor centre, and *a* is a limiting value of the deflection of the floor under 1 kN. The peak velocity of the floor can be written as:

3 $$v\le {b}^{\left({f}_{1}\zeta -1\right)} \left[{\text{m}}/({{\text{Ns}}}^{2})\right],$$

where *v* is the unit impulse velocity (maximum initial value of vertical floor velocity (m/s)) caused by a unit impulse *v* = 1 Ns applied at the point on the floor giving maximum response.$\zeta$ is the modal damping ratio (a typical value for timber floors can be 1–2%); *b* is the velocity response constant. Instructively, Eq. (3) is designed to simulate the effect of heel impact (jumping load) or oscillations with high-frequency components (8–40 Hz) on timber floors. The quantity ${b}^{\left({f}_{1}\zeta -1\right)}$ represents permissible floor velocity. As stated by EKS 10 (Swedish national application document), the following limiting values can be used: *a* = 1.5 mm/kN, *b* = 100 m/Ns^2^ to guarantee better performance^14^. These will be considered in this study as well as the value $\zeta$ = 0.01. Although this value of damping is quite low, it is taken here to have the calculation be on the safe side. Note, however, that there are other rules and design limits among European countries used elsewhere, e.g., in this British Annex to EC5, *a* = 1.8 mm for *L* ≤ 4000 mm and *a* = 16,500/*L*^1.1^ for *L* > 4000 mm and *b* = 180-60*a* for *a* ≤ 1 mm and *b* = 160-40*a* for *a* > 1 mm^15^. For example, for joist span *L* = 4.5 m, *a* = 1.6 mm and *b* = 97.

The value of *w* in Eq. (2) may be estimated using the following expression^14^:

4 $$w=\kappa \frac{F{L}^{3}}{48EI},$$

where *F* = 1 kN, *κ *is a distribution loading factor, which takes into account the bending stiffness (*EI*) of the joists in both directions of the floor; otherwise, this value sets to *κ* = 1.0. The value of *κ* can be obtained as:

5 $$\kappa =\left\{\begin{array}{c}-4.7{\beta }^{2}+2.9\beta +0.4 \,\,when\,\, 0\le \beta <0.3\\ 0.8+0.2\beta \,\,when\,\, 0.3\le \beta <1.0\end{array}\right.$$

where

6 $$\beta =\frac{{(EI)}_{L}}{{(EI)}_{B}}{\left(\frac{s}{L}\right)}^{4},$$

where (*EI*)_*L*_ is the bending stiffness of the floor (Nm^2^/m) in the stiffer direction (along joists) and (*EI*)_*B*_ is the bending stiffness of the floor (Nm^2^/m) in the direction perpendicular to the stiffer direction; *s* is the spacing between the joists and *L* is the span of the simply supported joists. Note, however, that there are other methods to estimate the vertical deflection of a timber floor: see e.g. Ref.^15^.

The peak velocity, *v,* in Eq. (3) for a rectangular floor system, simply supported on all four sides, can be expressed as Ref.^2^:

7 $$v=\frac{4\left(0.4+0.6{n}_{40}\right)}{mBL+200} ({\text{m}}/{{\text{Ns}}}^{2}) ,$$

where *n*_40_ is the number of first order modes with natural frequencies lower than 40 Hz and is given by:

8 $${n}_{40}={\left[\left({\left(\frac{40}{{f}_{1}}\right)}^{2}-1\right){\left(\frac{B}{L}\right)}^{4}\left(\frac{{\left(EI\right)}_{L}}{{\left(EI\right)}_{B}}\right)\right]}^{0.25} ,$$

where *B* is the floor width (m) and (*EI*)_*B*_ < (*EI*)_*L*_*.* Equation (3) is valid for *f*_1_ < 40 Hz, and vibrations modes with natural frequencies higher than 40 Hz are unimportant and can thus be disregarded^2^.

## Section properties

If the floorboards are nailed or screwed to the joists, the floor system will not have full interaction between the boarding and timber beams. A partial composite action between flooring and joists can thus be assumed. However, in this study, such a partial interaction is neglected. The reason is to guarantee the safety conditions in practice, since the human body is in general sensitive to vibrations. At any rate, it can be shown, that the results of partial interaction will not typically deviate significantly from what is considered in this study. This case will be treated in a forthcoming article. The bending stiffness (*EI*) of the floor system can therefore be calculated with respect only to the joists. If, on the other hand, the floorboards are glued effectively to the joists, the floor system will be assumed to have full interaction between the boarding and joists, and full composite action can thus be obtained. Consequently, the bending stiffness of the floor system will be calculated as with the case of glued thin flanged beam forming an equivalent composite T-section.

**Figure 2:**
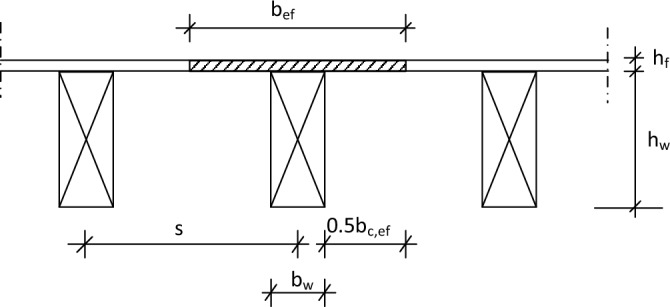
T-section beam.

The effective flange width of the flooring panel, *b*_*ef*_, is calculated as:

9 $${b}_{ef}={b}_{c,ef}+{b}_{w},$$

where *b*_*c,ef*_ is the effective flange width in compression and *b*_*w*_ is the joist width as illustrated in Fig. 2. The value of the effective flange width that can be assumed to interact in the T-section must not exceed certain values due to effects of shear lag and plate buckling; for OSB/3, where the main chip direction is parallel to the joist, it can be obtained as Ref.^16^:

10 $${b}_{c,ef}\le 25{h}_{f},$$

11 $${b}_{c,ef}\le 0.15L,$$

where *h*_*f*_ is the thickness of the floorboard, *L* is span of the joist. Additionally, *b*_*ef*_ cannot, technically, exceed the centre distance between joists, *s*:

12 $${b}_{ef}\le s.$$

The transformed flange width of the flooring panel into timber, *b*_*ftd*_, may be expressed as:

13 $${b}_{ftd}=\frac{{{(E}_{0,mean)}}_{OSB}}{{{(E}_{0,mean)}}_{joist}}{b}_{ef}.$$

Area of the transformed cross-section:

14 $$A={b}_{ftd}{h}_{f}+{b}_{w}{h}_{w}.$$

Based on Eqs. (13) to (14), the neutral axis depth (e.g. from the top face) and subsequently the second moment of area of the transformed T-section, can be calculated.

From an instructive point of view, in order that the gluing or bond between floorboards and joists shall be effective, the timber joists must be planed (or surfaced), cleaned before applying glue, and adhesive pressure is obtained through screws (with at least a length of 50 mm) distributed at 300 mm (at floorboards’ edge, the distance is halved)^14,17^. It is worthwhile to mention here that the planar or rolling shear at the flange glue lines needs to be checked to satisfy the requirements for the ultimate limit state^16^.

## Results and discussion

**Figure 3:**
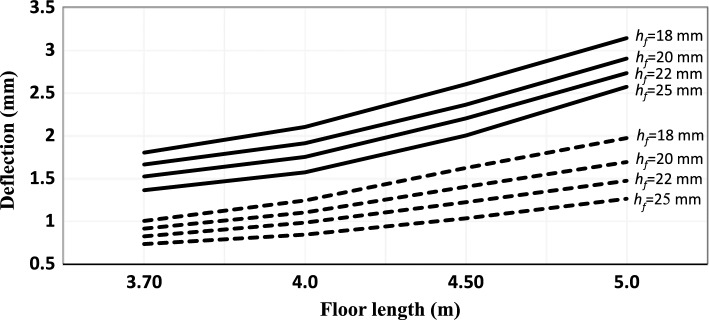
Deflection of the floor construction versus floor length (joist span) for joist spacing 600 mm. ▬▬▬▬ Nailed/screwed floorboards, **‐ ‐ ‐ ‐ ‐ ‐** glued floorboards.

**Figure 4:**
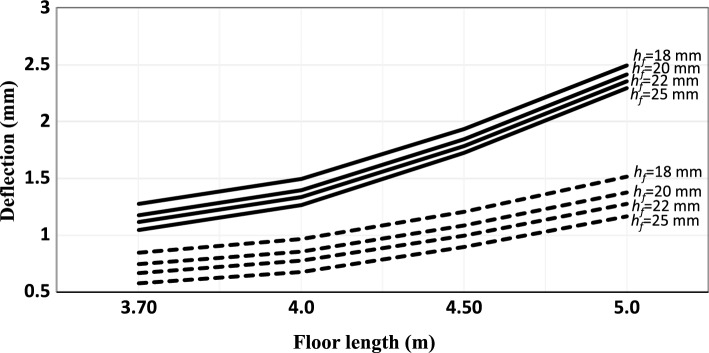
Deflection of the floor construction versus floor length (joist span) for joist spacing 400 mm. ▬▬▬▬ Nailed/screwed floorboards, **‐ ‐ ‐ ‐ ‐ ‐** glued floorboards.

**Figure 5:**
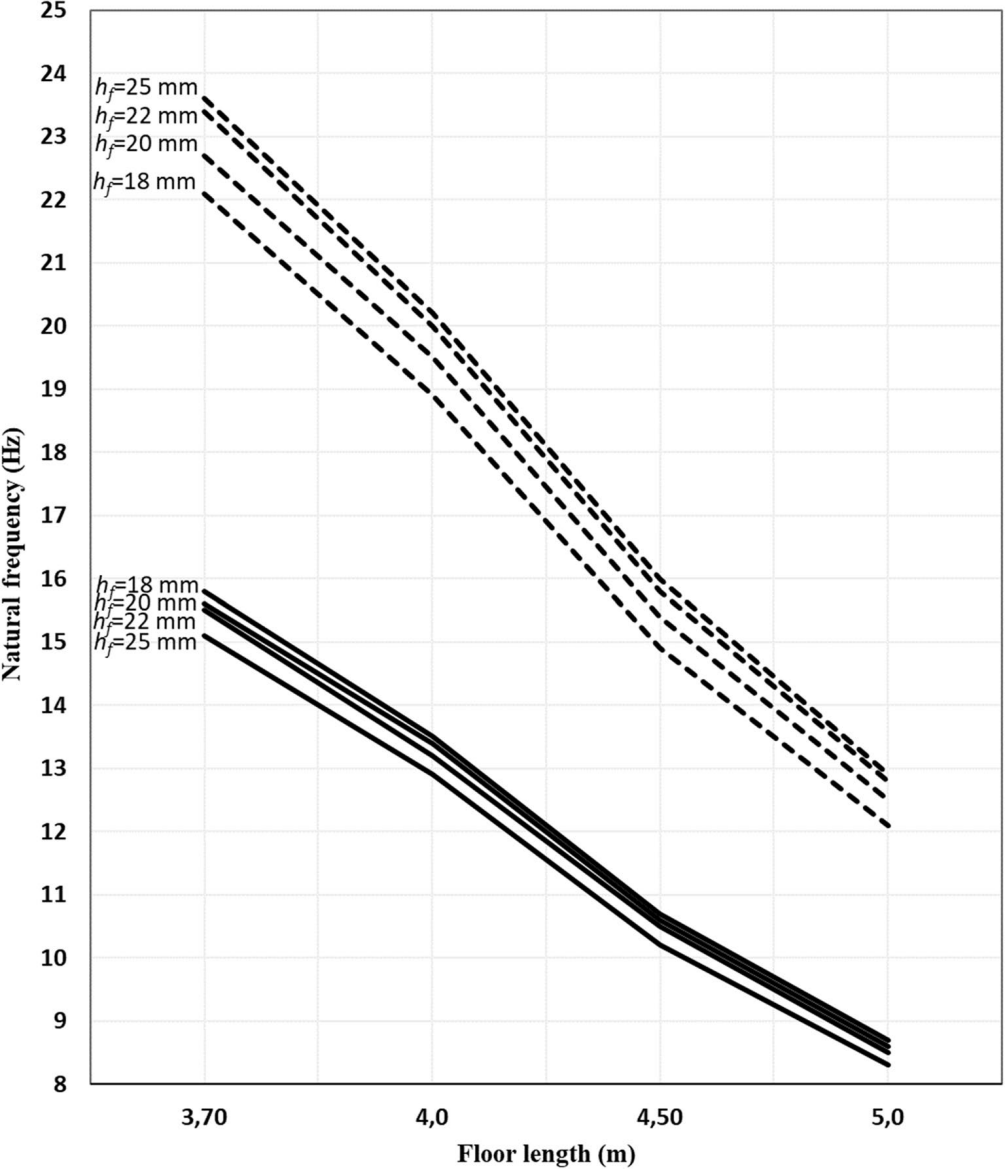
Natural frequency of the floor construction versus floor length (joist span) for joist spacing 600 mm. ▬▬▬▬ Nailed/screwed floorboards, **‐ ‐ ‐ ‐ ‐ ‐** glued floorboards.

**Figure 6:**
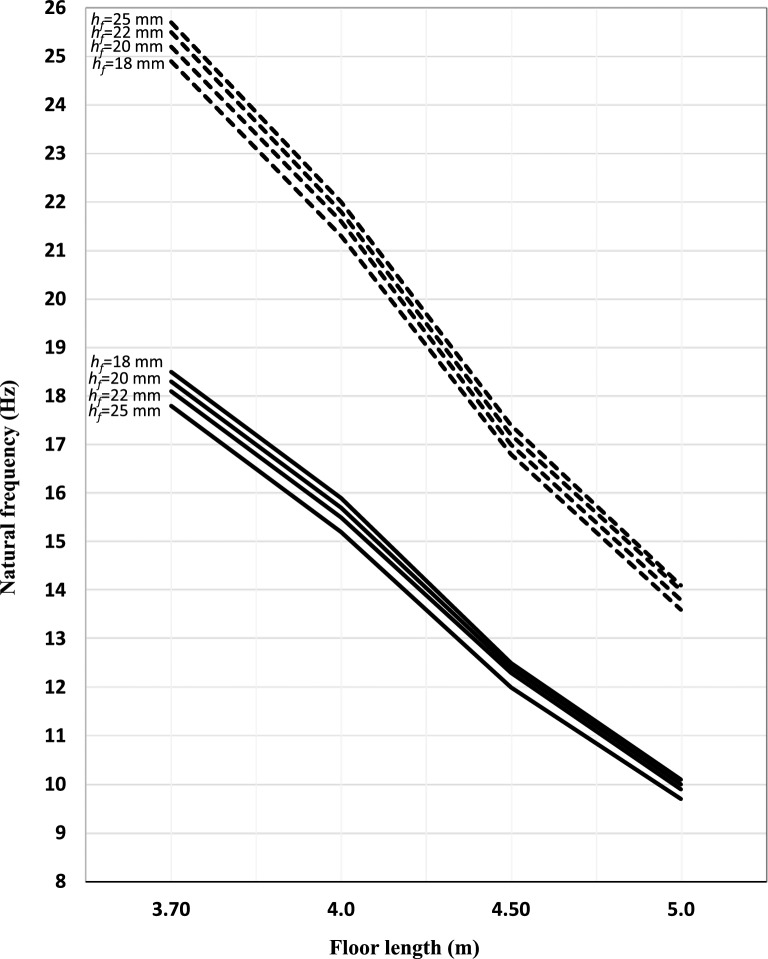
Natural frequency of the floor construction versus floor length (joist span) for joist spacing 400 mm. ▬▬▬▬ Nailed/screwed floorboards, **‐ ‐ ‐ ‐ ‐ ‐** glued floorboards.

The results of parameter study of the factors that affect the vibration acceptability of floor structure are presented in Tables 3, 4, 5 and 6 and Figs. 3, 4, 5 and 6.

**Table 3:** Results of design check of floor vibration for floor 5 m × 3.7 m.

Parameter	Natural frequency, *f*_1_, Eq. (1), (Hz)	Deflection *w*, Eq. (4), (mm)	Peak velocity; permissible floor velocity (*v*; ${b}^{\left({f}_{1}\zeta -1\right)}$), Eq. (3), (mN^–1^ s^–2^)	Requirements satisfied?
Nailed/screwed floorboards				
Thickness,* h*_*f*_ = 18 mm				
* s* = 600 mm	15.8	1.80	(0.023, 0.021)	No
* s* = 400 mm	18.5	1.27	(0.022, 0.023)	Yes
* h*_*f*_ = 20 mm				
* s* = 600 mm	15.6	1.66	(0.021, 0.021)	No
* s* = 400 mm	18.3	1.17	(0.020, 0.023)	Yes
Thickness, *h*_*f*_ = 22 mm				
* s* = 600 mm	15.5	1.52	(0.020, 0.020)	Yes
* s* = 400 mm	18.1	1.11	(0.018, 0.023)	Yes
Thickness, *h*_*f*_ = 25 mm				
* s* = 600 mm	15.1	1.36	(0.018, 0.020)	Yes
* s* = 400 mm	17.8	1.04	(0.017, 0.023)	Yes
Glued floorboards				
Thickness, *h*_*f*_ = 18 mm				
* s* = 600 mm	22.1	1.00	(0.022, 0.028)	Yes
* s* = 400 mm	24.9	0.84	(0.020, 0.031)	Yes
Thickness, *h*_*f*_ = 20 mm				
* s* = 600 mm	22.7	0.91	(0.020, 0.029)	Yes
* s* = 400 mm	25.2	0.74	(0.019, 0.032)	Yes
Thickness, *h*_*f*_ = 22 mm				
* s* = 600 mm	23.4	0.82	(0.019, 0.029)	Yes
* s* = 400 mm	25.5	0.66	(0.017, 0.032)	Yes
Thickness, *h*_*f*_ = 25 mm				
* s* = 600 mm	23.6	0.73	(0.017, 0.030)	Yes
* s* = 400 mm	25.7	0.57	(0.015,0.033)	Yes

**Table 4:** Results of design check of floor vibration for floor area 5 m × 4 m.

Parameter	Natural frequency, *f*_1_, Eq. (1), (Hz)	Deflection *w*, Eq. (4), (mm)	Peak velocity; permissible floor velocity (*v*; ${b}^{\left({f}_{1}\zeta -1\right)}$ ), Eq. (3), (mN^–1^ s^–2^)	Requirements satisfied?
Nailed/screwed floorboards				
Thickness,* h*_*f*_ = 18 mm				
* s* = 600 mm	13.5	2.10	(0.022, 0.019)	No
* s* = 400 mm	15.9	1.49	(0.021, 0.021)	Yes
* h*_*f*_ = 20				
* s* = 600 mm	13.4	1.91	(0.020, 0.019)	No
* s* = 400 mm	15.7	1.39	(0.019, 0.021)	Yes
Thickness, *h*_*f*_ = 22 mm				
* s* = 600 mm	13.2	1.75	(0.019, 0.018)	No
* s* = 400 mm	15.5	1.33	(0.018, 0.020)	Yes
Thickness, *h*_*f*_ = 25 mm				
* s* = 600 mm	12.9	1.57	(0.017; 0.018)	No
* s* = 400 mm	15.2	1.26	(0.016, 0.020)	Yes
Glued floorboards				
Thickness, *h*_*f*_ = 18 mm				
* s* = 600 mm	18.9	1.24	(0.021, 0.024)	Yes
* s* = 400 mm	21.3	0.96	(0.020, 0.027)	Yes
Thickness, *h*_*f*_ = 20 mm				
* s* = 600 mm	19.5	1.10	(0.020, 0.025)	Yes
* s* = 400 mm	21.6	0.85	(0.018, 0.027)	Yes
Thickness, *h*_*f*_ = 22 mm				
* s* = 600 mm	20.0	0.98	(0.018, 0.025)	Yes
* s* = 400 mm	21.8	0.77	(0.017, 0.027)	Yes
Thickness, *h*_*f*_ = 25 mm				
* s* = 600 mm	20.2	0.84	(0.016, 0.025)	Yes
* s* = 400 mm	22.0	0.67	(0.015, 0.028)	Yes

**Table 5:** Results of design check of floor vibration for floor area 5 m × 4.5 m.

Parameter	Natural frequency, *f*_1_, Eq. (1), (Hz)	Deflection *w*, Eq. (4), (mm)	Peak velocity; permissible floor velocity (*v*; ${b}^{\left({f}_{1}\zeta -1\right)}$ ), Eq. (3), (mN^–1^ s^–2^)	Requirements satisfied?
Nailed/screwed floorboards				
Thickness,* h*_*f*_ = 18 mm				
* s* = 600 mm	10.7	2.60	(0.020, 0.016)	No
* s* = 400 mm	12.5	1.93	(0.019, 0.018)	No
* h*_*f*_ = 20				
* s* = 600 mm	10.6	2.36	(0.019, 0.016)	No
* s* = 400 mm	12.4	1.84	(0.018, 0.018)	No
Thickness, *h*_*f*_ = 22 mm				
* s* = 600 mm	10.5	2.20	(0.017, 0.016)	No
* s* = 400 mm	12.3	1.78	(0.016, 0.018)	No
Thickness, *h*_*f*_ = 25 mm				
* s* = 600 mm	10.2	2.00	(0.015, 0.016)	No
* s* = 400 mm	12.0	1.72	(0.014, 0.017)	No
Glued floorboards				
Thickness, *h*_*f*_ = 18 mm				
* s* = 600 mm	14.9	1.62	(0.020, 0.020)	No
* s* = 400 mm	16.8	1.20	(0.019, 0.022)	Yes
Thickness, *h*_*f*_ = 20 mm				
* s* = 600 mm	15.4	1.40	(0.018, 0.020)	Yes
* s* = 400 mm	17.0	1.08	(0.017, 0.022)	Yes
Thickness, *h*_*f*_ = 22 mm				
* s* = 600 mm	15.8	1.22	(0.017, 0.021)	Yes
* s* = 400 mm	17.2	0.99	(0.016, 0.022)	Yes
Thickness, *h*_*f*_ = 25 mm				
* s* = 600 mm	16.0	1.03	(0.015, 0.021)	Yes
* s* = 400 mm	17.4	0.89	(0.014, 0.022)	Yes

**Table 6:** Results of design check of floor vibration for floor area 5 m × 5 m.

Parameter	Natural frequency, *f*_1_, Eq. (1), (Hz)	Deflection *w*, Eq. (4), (mm)	Peak velocity; permissible floor velocity (*v*; ${b}^{\left({f}_{1}\zeta -1\right)}$ ), Eq. (3), (mN^–1^ s^–2^)	Requirements satisfied?
Nailed/screwed floorboards				
Thickness,* h*_*f*_ = 18 mm				
* s* = 600 mm	8.7	3.14	(0.019, 0.015)	No
* s* = 400 mm	10.1	2.49	(0.018, 0.016)	No
* h*_*f*_ = 20 mm				
* s* = 600 mm	8.6	2.90	(0.017, 0.015)	No
* s* = 400 mm	10.0	2.41	(0.016, 0.016)	No
Thickness, *h*_*f*_ = 22 mm				
* s* = 600 mm	8.5	2.73	(0.016, 0.015)	No
* s* = 400 mm	9.9	2.35	(0.015, 0.016)	No
Thickness, *h*_*f*_ = 25 mm				
* s* = 600 mm	8.3	2.57	(0.014, 0.015)	No
* s* = 400 mm	9.7	2.29	(0.013, 0.016)	No
Glued floorboards				
Thickness, *h*_*f*_ = 18 mm				
* s* = 600 mm	12.1	1.97	(0.018, 0.017)	No
* s* = 400 mm	13.6	1.51	(0.017, 0.019)	No
Thickness, *h*_*f*_ = 20 mm				
* s* = 600 mm	12.5	1.69	(0.017, 0.018)	No
* s* = 400 mm	13.8	1.37	(0.016, 0.019)	Yes
Thickness, *h*_*f*_ = 22 mm				
* s* = 600 mm	12.8	1.47	(0.016, 0.018)	Yes
* s* = 400 mm	14.0	1.27	(0.015, 0.019)	Yes
Thickness, *h*_*f*_ = 25 mm				
* s* = 600 mm	12.9	1.26	(0.014, 0.018)	Yes
* s* = 400 mm	14.1	1.16	(0.013, 0.019)	Yes

### Nailed/screwed floorboards

The thickness of floorboards (*h*_*f*_) is inversely proportional to the resonant (natural) frequency (*f*_1_) and has in general a minimal effect, as shown in Figs. 5, 6. The difference in natural frequencies between thicknesses 18 mm and 25 mm equal to about 1 Hz for short span lengths, and it decreases gradually as floor length increases to be equal, approximately, to 0.5 Hz. This difference is observed irrespective of spacing between joists. Since there is no interaction assumed to exist between the boarding and joists, the thickness of the floorboards only affects the mass of the floor and subsequently does not contribute significantly to the natural frequency of floor as inspected from Eq. (1). As the changes in the stiffness to mass ratios are not only affected by floor thickness but also by the span between joists, the natural frequencies for floors with dense distribution of joists and thicker boards will be higher than natural frequencies for floors with sparse distribution of joists and slender boards. Although thicker boards are more robust and sustainable to greater loads, they result in decreasing the natural frequency of the floor. Additionally, as can be seen in Figs. 5 and 6, short span floors possess higher natural frequency of about 15–19 Hz while long span floor possess 8–10 Hz.

Let us, with the help of Eq. (1), define a new quantity: critical length, *L*_*cr*_ as the length in which the floor’s natural frequency is equal to 8 Hz:

15 $${L}_{cr}=0.443\sqrt[4]{\frac{{\left(EI\right)}_{L}}{m}}.$$

Equation (15) may further be simplified for the case of non-composite action between boarding and joists to:

16 $${L}_{cr}=0.238\sqrt[4]{\frac{Eb{h}^{3}}{sm}.}$$

Accordingly, for floor span lengths over critical length (*L* > *L*_*cr*_), the natural frequency is obtained as *f*_1_ < 8 Hz, which is not preferable in principle, and the situation will thus need special investigations, as mentioned earlier. Subsequently, the critical length is dependent here on the mass of the floor and joist spacing for nailed/screwed floorborads: for *s* = 600 mm, the interval range of the critical lengths varies within *L*_*cr*_ = 5.2 m − 5.1 m (*h*_*f*_ = 18 mm − *h*_*f*_ = 25 mm) and for *s* = 400 mm, *L*_*cr*_ = 5.76 m − 5.63 m (*h*_*f*_ = 18 mm − *h*_*f*_ = 25 mm). Consequently, by decreasing the spacing between joists, the critical length will increase. This effect can subsequently be utilized as a practical measure to satisfy the requirements of vibration comfort.

With respect to deflection criteria, the thickness of floorboards is inversely proportional to the deflection of the floor, see Figs. 3 and 4. On the other hand, the joist spacing is directly proportional to the deflection of the floor. This is mainly because of the effect of bending stiffness. Further, as inspected from Eq. (5), as the joist span increases, the deflection is also increasing.

With respect to the criterion of peak velocity of the floor that simulates the high-frequency components of the walking vibrations, one can note that it is closely related to the deflection of the floor. For all the treated cases, this criterion is satisfied once the deflection criterion is satisfied. Additionally, it is noted that increasing the thickness of floorboards will decrease the peak velocity, and with thickness *h*_*f*_ = 25 mm, it seems that this criterion is satisfied irrespective of floor length.

With respect to satisfying all requirements for vibration comfort, it is noted that, as floor length increases, these are not satisfied irrespective of the floorboard’s thicknesses. This situation is evident for floor lengths 4.5 m and 5 m due to large deflections and peak velocities of the floors.

### Glued floorboards

In general, the glued floorboards perform much better than nailed/screwed floorboards due to the composite action and consequently higher stiffness of the T-section. Against this background, greater increased natural frequency and reduced floor deflection are observed.

With respect to the deflection of the floor, the same conclusions drawn previously for the nailed/screwed floorboards are valid here, as can be seen in Figs. 3 and 4. However, the deflection is significantly lower in this case; a decrease of about 60% compared to the nailed/screwed floorboards is obtained for the different thicknesses investigated in this study. By gluing the floorboards effectively implies adding stiffness, which results in increasing the natural frequency and decreasing the deflection.

With respect to the natural frequency, a notable result here is that the thickness of floorboards is directly proportional to the resonance (natural) frequency and has, in general, some effect, depending on the spacing between joists. Such behaviour is not observed for the nailed/screwed floorboards. The difference in natural frequencies between thicknesses 18 mm and 25 mm (for *s* = 600 mm) is equal to about 1.5 Hz for short span lengths, and it decreases gradually as floor length increases to be equal to 1 Hz. However, as the spacing between joist decreases, this difference will gradually resemble the situation for nailed/screwed floorboards.

As with the case of nailed/screwed floorboards, the natural frequency of the floor system is directly proportional with the spacing between joists and inversely proportional with the floor length.

The peak velocity of the floor is directly proportional with deflection of the floor system, as with the case of nailed/screwed floorboards. Additionally, as the thickness of floorboards increases, the peak velocity decreases, and with thickness *h*_*f*_ = 20 mm, the criterion of peak velocity is satisfied irrespective of floor length. Consequently, there is no need to have thicker floorboards than the allowable limit to lower the peak velocities.

With respect to satisfying all requirements of vibration comfort, the results in Tables 3, 4, 5 and 6 show that in almost all cases, the floor system is adequate to vibration comfort except in four cases: *L* = 4.5 m (*h*_*f*_ = 18 mm, *s* = 600 mm) and *L* = 5 m (*h*_*f*_ = 18 mm and *h*_*f*_ = 20 mm, *s* = 600 mm). This suggests that the floorboard’s thicknesses: *h*_*f*_ > 20 mm and narrowly spread joists, *s* = 400 mm, are the best solution to satisfy the vibration requirements in this case.

### On the control of floor vibration

In almost all cases investigated in this study (Tables 3, 4, 5 and 6), the inadequacy of floor to vibration comfort is due to the deflection criteria, which simulate the low frequency components of the footstep’s oscillation. Technically, to overcome this decisive problem, the designer may decrease the joist spacing, use thicker floorboards and glued floorboards to increase the bending stiffness of the floor system. In the same context, adding more joists by reducing distance between adjacent joists will improve in general the floor serviceability pertaining to vibration comfort, as it increases the mass of the floor as well as longitudinal stiffness of the floor. Further, the mass of floor can also be increased by using concrete screed or parquet flooring and adding longitudinal stiffeners in the floor system.

Another practical measures to reduce floor vibration is by increasing the damping of the floor system by installing floor dampers: for instance, a dynamic vibration absorber^18^. Alternatively, the designer may choose materials that have higher damping ratios. To retrofit the floor construction by bridging, blocking and cross bracing can be an advantageous measure to increase the transversal stiffness in a floor^19^.

## Conclusion

In this study two types of floor systems are investigated based on the serviceability vibrational comfort of Eurocode 5. In the first system, the floorboards are nailed or screwed to the joist and no interaction is produced between them, and in the second system, the floorboards are glued sufficiently to the joist so that a full composite action is produced. The result suggests that glued floorboards perform much better with respect to natural frequency, static deflection and peak velocity than the nailed or screwed floorboards. In almost all cases of glued floorboards, the result complies fully with the Eurocode 5 design vibration requirements. However, as floor lengths increase, the static deflection in particular will increase beyond the allowable limit for relatively thin floor panels and relatively widely spread joists. For both cases, increasing floorboards thickness and decreasing the joist span by adding more beams can even yield better results to satisfy the requirement of vibration comfort.

An interesting finding in this study is that increasing the thickness of floorboards will have different effects on the resonant (natural) frequency of the floor system depending on the connection of floorboards to the load-bearing joists. If floorboards are glued, the resonant frequency will increase as panel thickness increases. If, on the other hand, floorboards are nailed or screwed, the resonant frequency will decrease as thickness increases. This is mainly due to the resulting stiffness and mass in both cases. Moreover, it is observed that by gluing the floorboards to the joist, the resonant frequency will increase by 40% to 60% more than the resonant frequency of the nailed/screwed floorboards. Additionally, for the floor systems investigated in this study, the fundamental frequency satisfies the criterion of 8 Hz, which implies that resonance with the frequency of walking will be avoided in this case.

In this study, the estimation of the floor mass accounts for the ceiling component and the acoustic material. If, on the other hand, these components are not considered (as in the case of bare floors) the mass of the floor will be lower. In this case, it can be shown that the dynamic behaviour pertaining to vibration comfort will not improv as inspected by Eqs. (1) and (3).

By introducing the concept of critical length of the floor length, a better picture of the resonant behaviour of the floor to improve floor serviceability can be obtained. As the floor lengths become higher than the critical length, the resonant frequency will become less than 8 Hz, which is not preferable (although special investigation is needed in this case) because it can result gradually in resonance between the floor vibration and human excitation.

To guarantee that glued floorboards in the floor system have an adequate performance in terms of vibrations, the gluing process must be carefully planned at the factory or in situ.

Technically, by screwing glued floorboards to the joist, one can provide tight connections and prevent possible floor squeaks that can be caused by movement at the connection between floorboards and joists. By adding glue to a screw hole, a stronger bond between the screw and the wood can be obtained, since the glue will fill any potential gaps or voids in the wood fibres.

For future studies, other parameters that can be investigated are the joist size and strength class, other types of floorboards than the OSB/3, effect of floor coverings, the effect of joist size and how dampers or damping materials can affect the vibration behaviour of the floor system in view of all criteria stated in the Eurocode 5.

## Data Availability

All data generated or analysed during this study are included in this published article in main manuscript file.
